# Development of a HIV-1 Virus Detection System Based on Nanotechnology

**DOI:** 10.3390/s150509915

**Published:** 2015-04-27

**Authors:** Jin-Ho Lee, Byung-Keun Oh, Jeong-Woo Choi

**Affiliations:** Department of Chemical & Biomolecular Engineering, Sogang University, #1 Shinsu-Dong, Mapo-Gu, Seoul 121-742, Korea; E-Mails: jino@sogang.ac.kr (J.-H.L.); bkoh@sogang.ac.kr (B.-K.O.)

**Keywords:** detection system, HIV-1 virus, electrical, electrochemical, optical, nanoparticle

## Abstract

Development of a sensitive and selective detection system for pathogenic viral agents is essential for medical healthcare from diagnostics to therapeutics. However, conventional detection systems are time consuming, resource-intensive and tedious to perform. Hence, the demand for sensitive and selective detection system for virus are highly increasing. To attain this aim, different aspects and techniques have been applied to develop virus sensor with improved sensitivity and selectivity. Here, among those aspects and techniques, this article reviews HIV virus particle detection systems incorporated with nanotechnology to enhance the sensitivity. This review mainly focused on four different detection system including vertically configured electrical detection based on scanning tunneling microscopy (STM), electrochemical detection based on direct electron transfer in virus, optical detection system based on localized surface plasmon resonance (LSPR) and surface enhanced Raman spectroscopy (SERS) using plasmonic nanoparticle.

## 1. Introduction

Viruses are one of the most infectious agents for destructive diseases related to living beings including animals and plants [1]. The threat mechanisms of viruses are diverse, but one common thing is that existing viruses require a host such as bacteria, plants, animals, and including humans to propagate their existence [2]. Most virus-related diseases, including flu and the common cold are exterminated by the host’s innate immune response. However, more serious diseases such as acquired immune deficiency syndrome (AIDS) avoid this mechanism and thus can threaten host survival. The major variant of AIDS is the well-known virus Human Immunodeficiency Virus (HIV), that affects the immune system to tolerate life-threatening infections [3]. From the date AIDS was recognized, intensive study has been performed on HIV to understand the phenomenon and to discover a cure for AIDS [4]. As a result, beside the main molecular structures of HIV particles, the replication factors and cycle, biochemical specificity and immunosuppressive properties have been identified [5].

At the early stage of HIV infection, the generic symptoms are often difficult to distinguish from those associated with the common cold or fevers. A detection system that can provide early viral detection would allow for prescribing more effective treatments. However, conventional detection including enzyme-linked immunosorbent assays (ELISA), polymerase chain reaction (PCR), and serologic tests systems are not sensitive enough, and moreover are time consuming and resource-intensive. For example, the ELISA system requires multiple steps and several agents with potential possibility for quenching [6]. Another powerful diagnostic system, PCR, that detects the nucleic fragments from sample solutions requires considerable sample preparation steps and can be easily interrupted by small inhibitors [7]. In addition, since infectious virus nucleic acid fragments present in the host after the infection has cleared or neutralized, PCR only can provide indirect detection of a virus infection [8,9]. Moreover, cell culturing is a time-consuming and labor intensive, and requires highly skilled persons to perform the process. In some cases, viruses cannot even be cultured at all [10] (Table 1). Therefore, highly sensitive, selective, fast, and easy to use virus detection systems are needed for more effective treatments.

**Table 1:** Comparison of sensitivity and limitations of conventional viral detection systems.

Detection System	Sensitivity (TCID_50_)	Limitations
ELISA	10^5^	Requires multiple sampling steps and reagents
PCR	5–100	Requires parallel testing of external standards, complex multistep manipulations [e.g., labor-intensive sample preparation (plasma separation and RNA extraction), amplification (expensive reagents), and detection]
Cell Culture	10^4^	Requires skilled technicians, time-consuming and costly. Some viruses cannot be cultured

Interest in nanotechnology has revealed new possibilities in developing more accurate and sensitive measurements of viruses based on the unique properties of nanoparticles, in particular those where the surface-to-volume ratio plays a vital role. The high surface-to-volume ratios of nanomaterials have led to their implementation in sensing systems since research on biosensor devices first began to engage with nanotechnology. The surface plasmon resonances of nanomaterials have also enriched the scope for developing novel sensor devices. On the other hand, bulk material sensors are less likely to benefit from extreme scaling. Advances in nanotechnology, bio/chemical synthesis, and thin film techniques have allowed material properties to be adapted to sensing platforms to provide enhanced performance.

In this paper we review and discuss recently developed HIV virus particle sensor systems integrated with nanotechnology, focused on electrical detection based on scanning tunneling microscopy (STM), electrochemical detection based on direct electron transfer in virus, and optical detection based on plasmonic nanoparticles (localized surface plasmon resonance: LSPR, surface enhanced Raman spectroscopy: SERS).

## 2. Electrical Detection System Based on Scanning Tunneling Microscopy

Electrical detection systems have emerged as a detection technique to measure biological events based on their merits of immediate response and cost effectiveness [11]. Moreover, the presence of a single virus particle was even measured by electrical detection methods in recent research and they were suggested as promising alternatives to traditional PCR or optic-based assays [12,13,14]. For example, Shafiee *et al*. have proposed a highly sensitive micro-electromechanical systems (MEMS) integrated device that utilizes impedance analysis of viral nano-lysates using HIV-1 and its multiple subtypes as a model system [14]. Viruses were captured through magnetic beads conjugated with gp120 antibodies and detected by impedance spectroscopy of the viral lysate based on change of the electrical conductivity of the sample. Moreover, the system was integrated with a microfluidic chip array system to reduce the complexity, sample preparation and amplification steps, which also allowed test to be operated with minimal training requirements and shortened the assay time. As a result, integration of microelectrodes into multi-array or microprocessor-controlled diagnostic tools established electrical sensing as a promising and powerful technology [15]. Such systems which utilize the electrical fingerprint of a pathogen in a sensitive electronic read-out could potentially open exciting avenues in developing practical diagnostic tools.

Apart from these, scanning tunneling microscopy (STM) has also attracted great interest among electrical technologies due to its useful high-resolution imaging properties. When a voltage is applied between a sharp metal probe and an electrically conductive material that is positioned approximately less than 10 nm away, a very small tunneling current is produced. According to quantum mechanics, this electrical current will pass through the surroundings without physical contact between the probe tip and surface. As the tip scans the conducting surface, a proportional change happens in the tunneling current with different density states, thus providing a real current profile of the surface.

**Figure 1:**
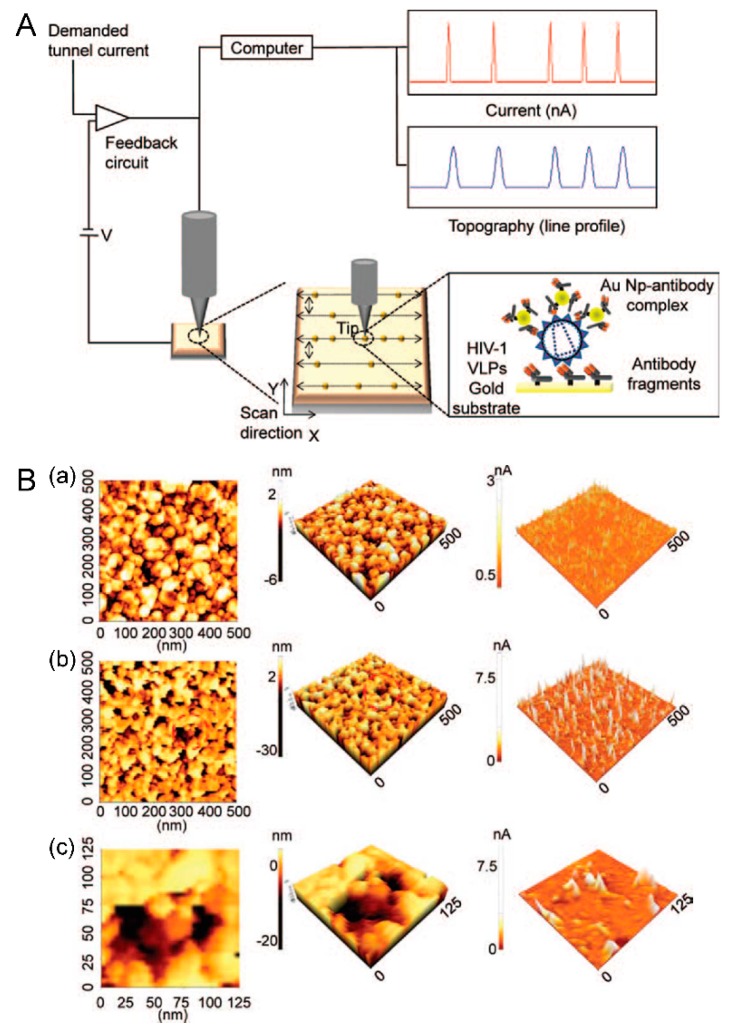
Vertical configured electrical detection system for HIV-1 virus. (**A**) Schematic diagram of an electrical detection system based on STM and its topography and the tunneling current profile of dispersed Au nanoparticle-antibody complexes based on immunoreactions; (**B**) Topographic images and current profile of: (**a**) bare gold surface and (**b**,**c**) biosurface fabricated with localized fragment antibody/target (HIV-1 VLPs)/Au nanoparticle-antibody complex. Figure reproduced with permission from [20], © 2014 American Scientific Publisher.

Several recent reports have described immunoassays based on STM, but these assays determine the presence of target analysts in a horizontal direction only [16,17,18,19]. In contrast, Lee *et al*. have demonstrated a vertically configured HIV virus sensor system based on STM (Figure 1) [20]. The basic concept was simple measurement of the abrupt change in tunneling current based on STM, which results from localized nanoparticles due to immunoreactions. The HIV virus particle was introduced to the Au surface functionalized by antibody fragments, and the Au nanoparticle-antibody complex was subsequently applied to form a sandwich-like configuration to localize the Au nanoparticles. Since the tunneling current responded to the tip-to-surface distance, localized Au nanoparticles cause a pulse-like current peak at each localization position. Therefore, the frequency of current peaks could be employed to quantify the amount of HIV virus particles based on the immunoreactions related to the Au nanoparticles present. Compare to the horizontally configured detection system, the vertically configured electrical detection system based on nanoparticles allows the measurement system to be extended for other viruses and other kind of pathogens either alone or as a multiple target assay with appropriate combinatorial design of detection probes within micro/nano dot arrays.

## 3. Electrochemical Detection Systems Based on Direct Electron Transfer from Virus Particles

In the electrochemical detection system for biomolecular interactions, it is well known that there should be an electro-active region which can undergo a redox reaction. Therefore, indirect detection methods based on DNA hybridization sandwich assays for biomolecules with additional electrochemical labels including redox enzyme complexes have been extensively developed (using horseradish peroxidase or alkaline phosphatase) [21,22], although it should be noted that even though these indirect electrochemical detection systems have attracted interest based on their virtues of high sensitivity and selectivity, they require more fabrication steps and are time consuming due to the additional labeling needed. In addition, since an enzyme is crucial to the functioning of the devices, enzyme-based indirect detection systems often suffer from poor reproducibility and unstable responses [23]. To overcome these problems, numerous researches have been performed to develop electrochemical detection systems based on direct electron transfer from biomolecules without using any mediators [24,25]. The direct electron transfer from a redox active biomolecule to an electrode could provide an easier way to develop label-free and sensitive detection systems. This method will simplify the detection system providing better stability by eliminating interfering reactions [26]. However, direct detection of the electrochemical signals generated by most redox biomolecules on conventional electrodes is a great challenge as a result of the fact the redox-active centers are usually deeply buried inside the biomolecules [27].

**Figure 2:**
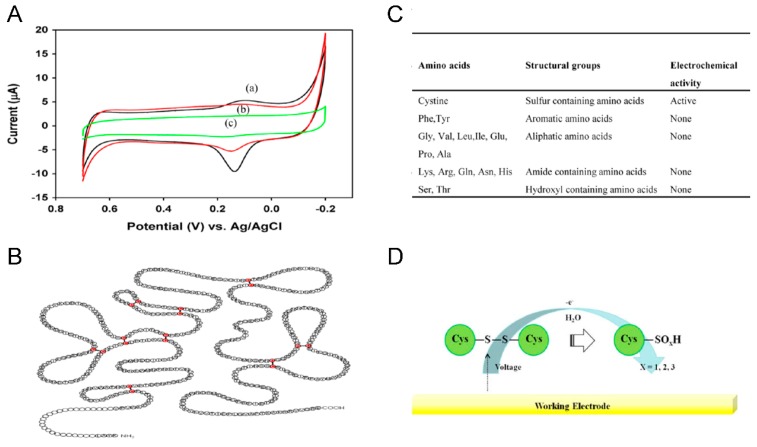
Electrochemical detection of HIV-1 virus based on direct electron transfer using Au nano dot A. Cyclic voltammogram of fabricated biosurface on an Au nanoparticle modified ITO electrode. (**A**) Bare Au nanoparticle modified ITO electrode; (**B**) fragmented Antibody; (**C**) HIV-1 VLPs, respectively, Schematic representation of B. the structure of gp120, C. expected electrochemical oxidation of disulfide bond and (**D**) Electrochemical activity of various amino acids. Figure reproduced with permission from [28], © 2013 Elsevier.

Interestingly, Lee *et al*. have measured the direct electron transfer from HIV virus particles for the first time [28]. The higher surface-to-volume ratio of modified three dimensional Au nanoparticles improved the surface area to provide better electron-transfer kinetics and higher background charging current, which made the electrode more sensitive to external influences. Based on these excellent electrical properties of modified Au nanoparticles, HIV-1 VLPs were successfully quantified by measuring the redox signal obtained from sulfur-containing cysteine residues located in the glycoprotein 120 of HIV virus particles. Since the proposed electrochemical detection system was designed for direct determination of HIV virus particles, the electrochemical communication between the redox target and electrode surface was able to reduce interference reactions and simplify the detection system with a small amount of reagent with better stability (Figure 2).

As known, electrochemical detection systems are inexpensive, robust, and highly sensitive. However, a labeling mediator was necessary for sensitive detection of bio/chemical analysts including viruses [29,30]. Apart from these drawbacks, the proposed detection system based on direct electron transfer from a virus suggests a new way to develop a highly sensitive label-free electrochemical biosensor for the determination of HIV-1 virus as well as other viral pathogens.

## 4. Optical Detection Systems Based on Plasmon Nanoparticles

Recent growth in the field of highly sensitive optical transducers has captured the interest and led to the development of various optical biosensors in diverse fields ranging from clinical diagnostics to therapy. Interestingly, recent highly advanced and newly developed optical detection systems are mostly based on the surface plasmon mechanism [31]. Surface plasmon resonance (SPR) is found in materials that have a negative (real) and small positive (imaginary) dielectric constant, especially in noble metals. When exposed to light, it is trapped near the surface as it interacts with the plasma of electrons near the metal surface. The resonant interaction between electron-charged oscillations near the surface of the metal and the electromagnetic field of the light creates the surface plasmon. Generally in surface plasmon-based detection systems, either propagating surface plasmon polarition or non-propagating localized surface plasmon resonance (LSPR) are used.

The surface plasmon polarition can be excited on the thin metal surface by light coupled with gratings or prisms [32]. This plasmon propagates along the metal surface and the dielectric interface until the energy is lost or absorbed [33]. Changes in the refractive index on the metal surface affect the plasmon resonance conditions, and can be measured by intensity changes, wavelength or angle shifts [34]. Comparably, when surface plasmons are confined to a nanomaterial which is much smaller than the wavelength of light, it is localized around the nanostructure with a specific frequency known as the LSPR. The spectral characteristics of the non-propagating LSPR are independent of size, shape, composition, and the local dielectric environment, therefore, the refractive index changes induced by an adsorbate on a metal nanostructures can be used to monitor molecular binding events [35,36,37].

**Figure 3:**
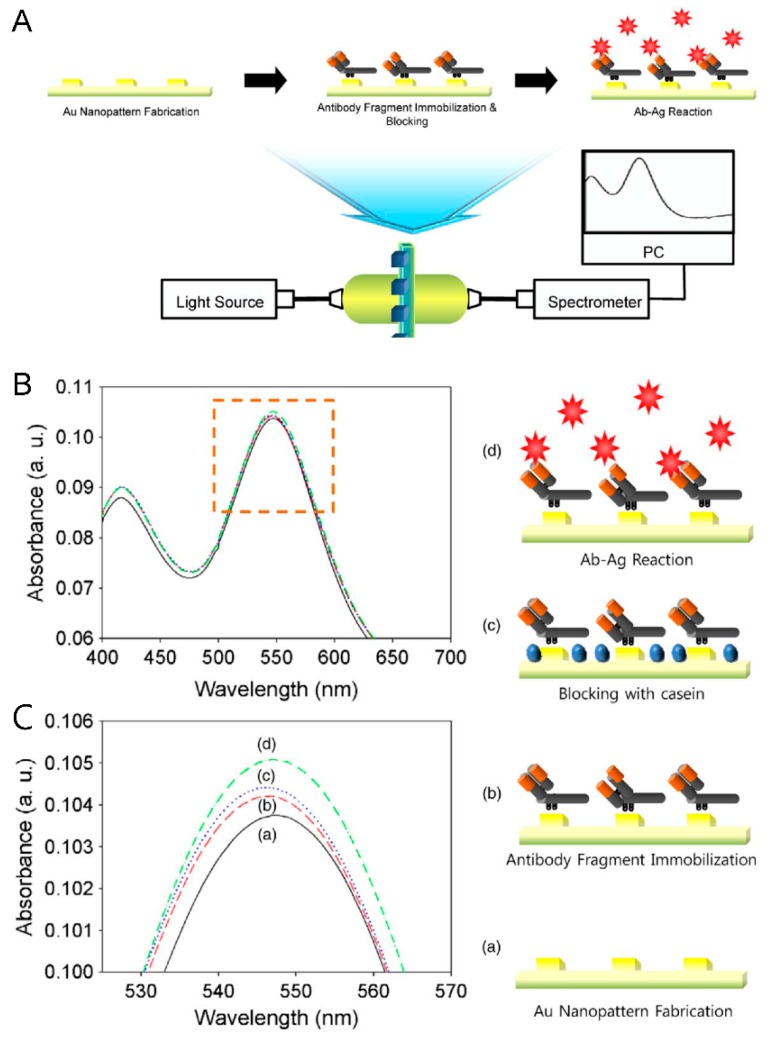
Optical detection of HIV-1 virus based on LSPR mechanism. (**A**) Schematic representation of the immunoassay configuration with LSPR method for the quantitative detection of HIV-1 particles; (**B**). Optical characteristics (change in absorbance peak) based on the: (**a**) bare Au nanopattern on ITO substrate; (**b**) fragmented antibody immobilized layer; (**c**) blocking with casein; (**d**) HIV-1 VLPs immuno reacted layer. Figure reproduced with permission from [40], © 2013 Elsevier.

A few studies on the development of detection systems based on the LSPR mechanism to detect viruses such as hepatitis B (HBV) and swine origin influenza A (H1N1) have been reported [38,39]. Lee *et al*. have proposed a simple electrochemical deposition technique to fabricate highly ordered circular shaped Au nanopatterns on a transparent indium tin oxide (ITO) substrate and applied it for HIV virus detection based on the LSPR mechanism [40] (Figure 3). As well, Demirci and his group have developed a more improved system with gold nanoparticles to capture, detect and quantify intact HIV including multiple subtypes (A, B, C, D, E, G, and subtype panel) from clinically discarded HIV-infected patient whole blood samples [41] and also showed the integration with a microfluidic system with an optical photonic crystal (a TiO_2_ coated polymer nanostructure) in a microwell system [42].

The presence of HIV virus particles was measured through absorbance changes, which resulted from the change in refractive index on the Au surface with specific binding occurring through immunoreactions without any labeling materials. Since optical detection systems based on the LSPR mechanism have various advantages such as rapid preparation, high sensitivity and selectivity, they could be a promising detection system to monitor and treat the disease.

Along with the non-propagating localized plasmon technique, surface-enhanced Raman spectroscopy (SERS) has been also been extensively studied in nanotechnology with increased interest. Currently, the mechanism models for SERS rely on electromagnetic (EM) field enhancement and chemical enhancement (CE) [43]. When incident light is irradiated onto metal nanostructures, the oscillating electric field drives an oscillation of the conducting electrons in the metal and results in an EM field enhancement. In a different way, when metals transfer charges via holes or electrons to adsorbed molecular bonds it increases the polarizability of the molecule and leads to CE [44,45]. Therefore, SERS assays can archive highly sensitive signals rapidly with low laser intensity and long wavelengths. Moreover, on account of the narrow band and ‘fingerprint’-like shape of the spectral signals, SERS can be applied to detect individual elements in multi-component samples [46]. The energy corresponding to each Raman frequency shift is related to the different rotational and vibrational transition states of the scattering molecules, thus the signal patterns of Raman spectra could represent the characteristic of a specific target molecule. In accordance to yhe high sensitivity and selectivity of the SERS technique, various researches has been performed to identify bio/chemical material composed of multi-component materials such as viruses, including bovine leukemia virus (BLV), respiratory syncytial virus (RSV) and HIV [47,48,49]. However, the development of SERS-active surface in a large area continues to face several problems such as a lack of uniformity and weak signal enhancement. In response to these problems, Lee and his coworkers have suggested a label-free HIV virus immunodetection system with a simpler process which can provide a large SERS-active surface area with Au nanodots [50]. While performing a simple electrochemical deposition, different percentages (w/v) of surfactant were applied to a gold precursor solution to obtain highly uniform SERS-active Au nanodot structures on a large surface area. On the highly ordered Au nanodots, the surface plasmon could be localized and the difference in dielectric constants functioned as the source of the electromagnetic enhancement, which work as the primary contributor to enhance the Raman signals. As a result of the excellent optical properties of highly ordered Au nanodots, HIV-1 virus particles were effectively determined by SERS without any labeling probes.

**Figure 4:**
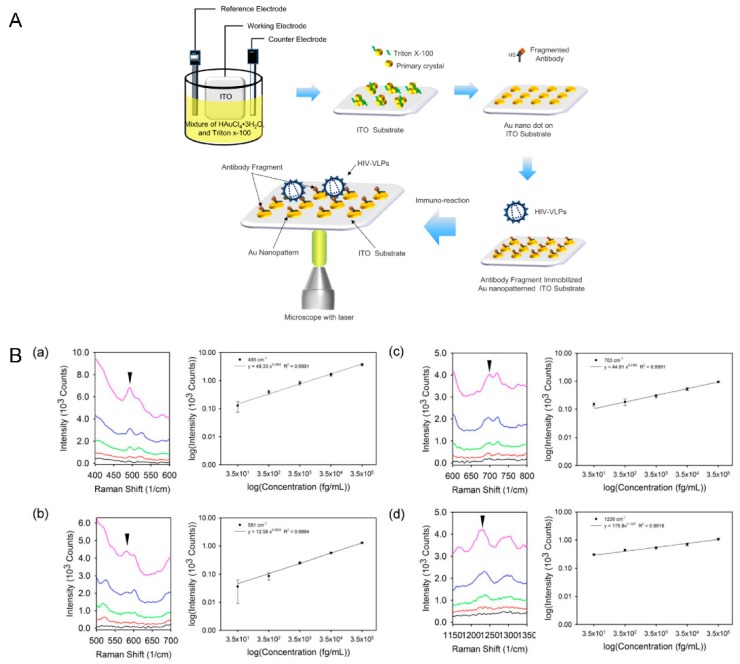
Optical detection of HIV-1 virus based on SERS. (**A**) Schematic representation of electrochemical deposition of uniformed Au nanodots and the immunoassay configuration using the SERS technique for the rapid and sensitive measurement of HIV-1; (**B**) The SERS spectra showed the changes in intensity of Raman peaks and linear plots related to different concentrations of HIV-1 VLPs. Figure reproduced with permission from [50], © 2015 American Scientific Publisher.

## 5. Outlook

Traditional viral detection methods are time consuming and expensive, and consequently, demand for accurate viral biosensors using rapid detection systems is increasing. Even though identifying the presence of viruses and viral components based on a biosensor (detection system) is a relatively new concept and for many of the techniques discussed this is still in its initial stages, along with the fact that the developed virus detection systems which have been discussed here also have several advantages and disadvantages. Firstly, in the electrical detection method based on STM, due to the vertical configuration, the proposed immunosensor system for the detection of surface proteins of virus may be applicable to other viruses and other kind of pathogens either alone or as a multiple-target analysis with appropriate combinatorial design of detection probes. In addition, since the detection system is based on electrical signaling, it could also possess a low detection limit. However, it has several disadvantages also such as being time consuming and requiring labeling material (conducting or semiconducting). Second, the detection systems based on direct electron transfer are simple to operate and labeling material is not necessary, but apart from that they have several disadvantages too. The sensitivity of the electrochemical signal without using an enzyme is very low and also the current flow is attenuated by insulating surfaces composed of biomaterials. In addition direct contact with electrolytes and the application of additional voltages to the target might lead to the degradation or denaturation of biomaterials. Moreover, till now, it is not completely defined where the redox reaction occurring. Thirdly, in the optical (Plasmon) detection methods based on LSPR mechanism, the ease of operation and high sensitivity offer the possibility of monitoring biological interactions simultaneously and provide a useful means to estimate the kinetic parameters. However, since in the LSPR detection system, the signal is mainly dependent on the refractive index around the metal structure, this method still requires great improvements to distinguish different binding events regarding multiple analysts. Combination with an analytical method such as Raman spectroscopy might however allow the identification of different viruses with improved detection limits. Finally, the optical (Plasmon) detection systems based on SERS have several advantages such as relatively lower laser intensity, longer wavelengths and rapid signal acquisition times. Moreover, unlike other vibrational spectroscopies, SERS can be conducted under ambient conditions and has a broad wavenumber range. Furthermore, it is able to diagnose the virus itself as well as its individual components due to its narrow band molecular spectra. In addition, the surface selectivity and sensitivity of SERS extends the capability to also develop a wide variety of interfacial systems. Despite the fact that the discussed detection systems promise various potential applications, interdisciplinary research efforts are still required to develop highly sensitive and reliable systems, which could replace currently available commercialized detection system (biosensors) and applications (Table 2).

In the near future, we believe that advances in the assembly of larger and more complex sensor arrays and their integration with nanoscale electronics and nano/microfluidic technologies will lead to exquisitely powerful detection systems which can be easily applied in the biomedical field [14,15]. A high throughput screening of viral pathogens will uncover vital information more clearly and help to understand the disease better to develop more efficient drugs for treatment.

**Table 2:** Comparison of four technologies based on their advantages and disadvantages.

Detection System	Advantages	Disadvantages
Electrical (STM)	Vertical configuration: Applicable to multiple analysis with appropriate combinatorial design of detection probes Low detection limit	Time consuming Requires labeling material (conducting or semiconducting)
Electrochemical (CV)	Simple to operate Labeling material is not necessary	Sensitivity of electrochemical signal without using enzyme is very low Current flow is attenuated by biomaterials Degradation or denaturation of biomaterials might occur Not completely defined where the redox reaction occurring
Optical (LSPR)	Ease of operation High sensitivity	Not able to distinguish different binding events regarding multiple analysts
Optical (SERS)	Requires relatively lower laser intensity and longer wavelengths Rapid signal acquisition times Can be conducted under ambient conditions Broad wavenumber range Narrow band spectra	Still requires interdisciplinary research effort to develop highly sensitive and reliable system such as integration with micro fluidic assay
